# Physicochemical and Sensory Properties of Davidson Plum (*Davidsonia jerseyana*) Sorbet, a Potential for New Functional Food Product

**DOI:** 10.3390/foods14162902

**Published:** 2025-08-21

**Authors:** Brittany Harriden, Costas Stathopoulos, Suwimol Chockchaisawasdee, Andrew J. McKune, Nenad Naumovski

**Affiliations:** 1 Discipline of Nutrition and Dietetics, Faculty of Health, University of Canberra, Canberra, ACT 2601, Australia; brittany.harriden@canberra.edu.au (B.H.); costas.stathopoulos@murdoch.edu.au (C.S.); suwimol.chockchaisawasdee@murdoch.edu.au (S.C.); 2 Functional Foods and Nutrition Research (FFNR) Laboratory, Singapore Institute of Technology, Singapore 828608, Singapore; andrew.mckune@canberra.edu.au; 3 University of Canberra Research Institute in Sport and Exercise (UCRISE), University of Canberra, Canberra, ACT 2601, Australia; 4 Food Futures Institute, Murdoch University, Nambeelup, WA 6150, Australia; 5 School of Medical, Molecular, and Forensic Sciences, Murdoch University, Murdoch, WA 6150, Australia; 6 Discipline of Biokinetics, Exercise and Leisure Sciences, School of Health Sciences, University of KwaZulu-Natal, Durban 4000, South Africa; 7 Discipline of Nutrition-Dietetics, Harokopio University, 17671 Athens, Greece; 8 Food, Chemical and Biotechnology Cluster, Singapore Institute of Technology, Singapore 828608, Singapore

**Keywords:** Australian native plants, Davidson plum, sorbet

## Abstract

The Australian native foods, despite high phytochemical composition, are severely underutilized in research and on the commercial market. One of these plants is the Davidson plum (*Davidsonia jerseyana*), a nutrient-dense and sustainable food ingredient. The study aimed to develop functional fruit sorbets incorporating freeze-dried Davidson plum powder (0–20% *w*/*w*) and evaluate their physicochemical, antioxidant, and sensory properties. Sorbets were created using strawberry, raspberry, pomegranate, and Davidson plum bases and analyzed for nutritional content, color, melting rate, texture, and antioxidant capacity (Total Phenolic Content (TPC), Total Flavonoid Content (TFC), Ferric Reducing Antioxidant Power (FRAP), Cupric Reducing Antioxidant Capacity (CUPRAC), 2,2-Diphenyl-1-picrylhydrazyl (Radical Scavenging Assay (DPPH)), total proanthocyanin and anthocyanin content. Sensory evaluation was also conducted using a semi-trained panel. The results showed that increasing Davidson plum concentration led to higher antioxidant activity and slower melting rates. Sorbets containing 10% and 15% Davidson plum demonstrated the highest levels of phenolic and flavonoid compounds. However, sensory analysis indicated that sorbets with 5% and 10% Davidson plum, particularly those made with a strawberry base were the most acceptable in terms of flavour, texture, and overall appeal. These findings suggest that incorporating Davidson plum into frozen desserts, especially at lower concentrations, can enhance both the functional and sensory qualities of sorbets while offering potential health benefits.

## 1. Introduction

Incorporating Australian native plants into modern dietary patterns may offer improved health outcomes and increase sustainability. Rediscovering Australian native foods and traditional methods is a strategy that can contribute to reducing the risk of chronic diseases (cardiovascular disease, type II diabetes mellitus (T2DM)), and Alzheimer’s disease [1,2]. Globally and in Australia, Indigenous communities have long utilised sustainable food practices, deeply linking their food to history, identity and the environment, a link which is integral to their holistic wellbeing [3]. Relatively recent research highlights the nutritional benefits of Australian native plants [2,4,5,6,7], their resilience to environmental stress, and their role in fostering sustainable food systems due to their high nutrient and phytochemical content [4,5,8]. Despite this, and in addition to increasing international interest in Australian native plants, the research, and utilisation of these plants into food products in Australia remains relatively unexplored. Numerous Indigenous fruits have not been investigated in Australia with only a few researched for commercial use [5,9,10]. There are several factors as to why, including ethical implications to industrialise Australian native plants, cultivation challenges, economic viability, and lack of awareness and promotion of the benefits and uses of Australian native plants among consumers and industries.

Australian Indigenous fruits such as the Davidson plum (*Davidsonia jerseyana, Davidsonia Pruriens*), Kakadu plum (*Terminalia ferdinandiana*), Finger lime (*Citrus australasica*) and Quandong (*Santalum acuminatum*) contain high quantities of different phytochemicals and polyphenols [5]. Polyphenol consumption was associated with being beneficial in reducing the risk of certain diseases such as chronic inflammation, T2DM, and other cardiovascular diseases [11,12]. The Davidson plum is a currently commercially grown and has been consumed by the Australian Indigenous communities as a raw fruit [13]. Presently, this fruit is used for a variety of food products including jams, chutneys, wine and commercial smoothies [4,14,15,16,17]. The Davidson plum contains a high and diverse polyphenolic profile, and it is also characterised by a unique flavour commonly described as earthy and pungent. Therefore, rather than using the fresh fruit, investigating Davidson plum powder as an ingredient has been suggested in the development of a functional food product, potentially providing health benefits [17].

The definition for functional foods and beverages is rather broad, however in general, these innovative products are designed to include additional bioactive ingredients with the potential of offering health-enhancing or disease-preventing benefits. The bioactive ingredients are included at levels that are both safe and adequate to deliver the intended effects [18]. The additional ingredients may include nutrients, dietary fiber, and variety of different phytochemicals [19]. Several studies have identified that fruits such as berries (blueberries, raspberries, strawberries) contain high amounts of polyphenols [20,21,22], while Davidson plum may contain up to three fold higher amounts of polyphenols than blueberries [5,13]. Thereby utilizing the Davidson plum to develop a functional food or beverage product may increase the overall nutrient capabilities and provide additional health benefits [23]. Previous studies have suggested that total polyphenolic intake of over 1 g/day (or greater than 100 mg of anthocyanins) consumed 2–3 times per day may be associated with potential health benefits [24,25]. Consuming these levels of polyphenols through a habitual dietary intake is hard to achieve. Therefore, the use of a functional food product containing quantifiable amounts of polyphenols and anthocyanins may be seen as an alternative pathway to reach physiologically relevant levels.

The development of a functional food or beverage products also requires the determination of physicochemical properties such as the textural, phytochemical and organoleptic properties. All of these properties are influenced by the composition, formulation and processing applications used during the product development [26]. Therefore, the aim of this study was to explore the use of Davidson plum powder, from an Australian native fruit with a unique flavour, as a functional ingredient in frozen dessert development. Davidson plum powder is utilised at different concentration ranges to examine the physicochemical, antioxidant and organoleptic properties of the newly developed products. Due to the unique flavour of the Davidson plum, strawberry, raspberry, and pomegranate were selected as a carrier base sorbet in addition to their nutritional profile [20,27,28,29].

## 2. Materials and Methods

### 2.1. Product Development

The frozen sorbet was selected as the food matrix of choice due to the low temperature, texture, and palatability. The fruit sorbets were developed based on previously published formulations from our team [30,31]. All ingredients, caster sugar, frozen fruits (raspberries and strawberries) and pomegranate juice were purchased from local supermarkets. Freeze-dried Davidson plum powder was purchased from a commercial supplier (Super Food Co., Oakleigh, VIC, Australia), citric acid was purchased from a commercial food supplier (Purewellness, Southport, QLD, Australia), and egg white albumen powder was purchased from Bulk nutrients (Hobart, TAS, Australia). The egg white albumen powder was used to provide structural stability, rigidity, and shape retention to the food product [32]. The sorbets were made using a commercial ice cream maker (Breville Pty Ltd., Sydney, NSW, Australia) and stored in a food-grade air-tight clean plastic containers in the freezer at −18 °C.

### 2.2. Sorbet Samples

Four flavours of sorbets were prepared (Davidson plum powder, pomegranate juice, frozen strawberry, and frozen raspberry). Each flavour contained different concentrations of freeze-dried Davidson plum powder (0, 5, 10, 15 and 20% weight/weight (*w*/*w*)), giving a total of 20 sorbet samples.

### 2.3. Macronutrient Analysis

The nutritional composition profiles of each sorbet were analysed using FoodWorks^®^ (v10; Xyris Software, Brisbane, QLD, Australia) nutrient analysis software.

### 2.4. Colour Analysis

The colour was analysed using a hand-held colorimeter (Konica Minolta Sensing Inc., Osaka, Japan)) with the L*, a*, b* values determined at ten randomly selected positions [31]. The final L*, a*, b* values were collated and represented as a mean value.

### 2.5. Melting Rate

The melting rate was evaluated in triplicates following previously published methods [31]. Briefly, frozen sorbets (20 ± 1 g) were placed on a wire rack (mesh size 1 cm^2^) in a temperature-controlled room (21  ±  0.5 °C) [33]. Measurements of the dripped volume were recorded at 5 min intervals up until 120 min. The drip volume (ml) was plotted against time (min), and melting rate determined and expressed as ml/min [33]. All samples were analysed in triplicates and results are represented as Mean ± SD value.

### 2.6. Texture Analysis

The texture analysis was performed using a texture analyser (TX-700, Lamy Rheology, Champagne au Mont d’Or, France) equipped with a 2 kg load cell and a P10 cylindrical probe (Diameter: 10 mm, Height: 40 mm; Ref: 130124). The hardness was measured through a compression test using sorbet in a 70 mL container left on the bench (21.8 ± 1.58 °C) for 30 min before measuring. The probe was moved at a speed of 3.5 mm/s from the surface until a depth of 10 mm. The force and time spent on penetration were recorded. All samples were analysed in triplicates and results are represented as Mean ± SD value.

### 2.7. Phytochemical Composition

All chemicals, 2,2-diphenyl-1-picrylhydrazyl (DPPH), sodium nitrite, sodium phosphate 6-hydroxy-2,5,7,8-tetramethylchroman-2-carboxylic acid (Trolox), copper(II) chloride dehydrate, ammonium acetate, ammonium molybdite, neocuprine, potassium persulfate, sodium acetate trihydrate, iron (III) chloride hexahydrate, Folin Ciocalteu reagent, sodium carbonate, gallic acid, aluminium chloride, sodium hydroxide, catechin, acetic acid, 2,4,6-Tris(2-pyridyl)-s-triazine (TPTZ), methanol, hydrochloric acid and sulfuric acid were purchased from Sigma Aldrich (Castle Hill, Sydney, NSW, Australia).

### 2.8. Methanolic Extraction

Total phenolic, flavonoid and antioxidant capacity were determined in methanolic extracts of the sorbet flavours following adapted method [34]. The extraction of antioxidants was completed by mixing the sorbet sample (0.5 g) with methanol (70%) at a 1:10 (*w*/*v*) ratio and sonicating (PowerSonic 420, Hwashin Technology, Seoul, Korea) in a water bath (26 °C) at maximum power for 30 min. The samples were then centrifuged (Sigma K4-12, Sigma Laborzentrifugen GmbH, Osterode am Harz, Germany) for 15 min (1800 g). The supernatants were stored in at −18 °C until analysis.

### 2.9. Total Phenolic Content

The total phenolic content for the sorbet was conducted using a modified method [35]. The absorbance was read at 765 nm and compared to a linear gallic acid standard curve and results were expressed as mg gallic acid equivalents (mg_GAE_). All samples were analysed in triplicates and results are represented as Mean ± SD value.

### 2.10. Total Flavonoid Content

Total flavonoid content of the sorbet was determined using a modified method by Petry et al. [36]. The absorbance was measured at a wavelength of 515 nm and a linear standard curve of the (±)-catechin hydrate concentrations against absorbance was generated to quantify the total flavonoid content in the samples. All results are expressed as mg catechin equivalents (mg_CE_). All samples were analysed in triplicates and results are represented as Mean ± SD value.

### 2.11. Anthocyanin Content

The measurement of total monomer anthocyanins within plants is used to determine the colour quality and can be determined using the pH differential method by Ali et al. [2]. The absorbances were recorded at 520 and 700 nm spectrophotometrically. The anthocyanin content was calculated using the equation below and values were represented in mg per g of cyanidin 3-glucoside (mg/g). All samples were analysed in triplicates and results are represented as Mean ± SD value.

TMAC (mg/g) = A × MW × DF /ε × l

where A = (A510 − A700 nm) pH 1 − (A510 nm − A700 nm) pH 4.5; MW = 449.2 g/mol; DF = dilution factor; ε = extinction coefficient of cyanidin-3-glucoside (26,900 L/cm mol).

### 2.12. Total Proanthocyanidins Content

Total proanthocyanidins content for the sorbet was determined using the vanillin method described by Ali et al. [2]. The sample solution of 25 µL was mixed with 150 µL of vanillin solution (4%). Then 25 µL of 32% H_2_SO_4_ was added into the mixture and incubated for 15 min at 25 °C. The absorbance was read at 500 and the standard curve of catechin (0–1000 μg/mL) and results are expressed as g catechin equivalents (μg_CE_/g). All samples were analysed in triplicates and results are represented as Mean ± SD value.

### 2.13. 2,2-diphenyl-1-picrylhydrazyl (DPPH) Assay

The DPPH analysis of sorbets using a DPPH assay based on previously published methods by Thaipong et al. [34]. The absorbance of the samples and standards were measured at a wavelength of 515 nm. A linear standard curve of the Trolox standard concentrations plotted against the absorbance and results were expressed as micromoles of Trolox equivalents per gram (μM_TE_). All samples were analysed in triplicates and results are represented as Mean ± SD value.

### 2.14. Ferric Reducing Antioxidant (FRAP) Assay

The FRAP analysis of the sorbet was based on the previously published method by Thaipong et al. [34]. The absorbance was measured at 593 nm and linear standard curve of the Trolox standard concentrations plotted against the absorbance and results were expressed as micromoles of Trolox equivalents per gram (μM_TE_). All samples were analysed in triplicates and results are represented as Mean ± SD value.

### 2.15. Cupric Ion Reducing Antioxidant Capacity (CUPRAC) Assay

The CUPRAC analysis of the sorbets was done using a method by Apak et al. [37]. The absorbance was measured at 450 nm and linear standard curve of the Trolox standard concentrations was plotted against the absorbance and results were expressed as micromoles of Trolox equivalents per gram (μM_TE_). All samples were analysed in triplicates and results are represented as Mean ± SD value.

### 2.16. Taste Evaluation and Product Acceptability

This study was approved by the Human Research Ethics Committee of the University of Canberra (HREC: 13368) and all participants have provided signed written informed consent prior to commencement of the study. For the sensory evaluation, 12 healthy participants aged between 18 and 62 years old (Males  =  5; Females  =  7), were recruited. This number is derived from the previous studies of similar type where trained panellists are used in the evaluation of different food products. All participants were screened against the eligibility criteria based on the self-reported health questionnaire. Participants were excluded if they had any food allergies, sensory sensitivity (including taste alternations due to recent COVID-19), liver disorders, liver conditions, gastrointestinal disorders, kidney disorders, history of smoking in the last 12 months, or if they are living with type 1 or 2 diabetes mellitus.

Participants were invited to attend the training session based on the sensory analysis evaluation procedures using established lexicons specifically orientated towards the sorbet, and products that contain Australian native ingredients [38,39]. Participants used a generated set of descriptive terms of native food products to evaluate the sorbets [38]. Each participant was provided with 5 g of control sorbets (Strawberry, Raspberry, Davidson plum and Pomegranate) that does not contain any Davidson plum powder, and one sorbet from each of the fruits that contains 20% (*w*/*w*) of the Davidson plum powder. Participants were asked to consume each of the sorbets and clean the pallet using unsalted crackers and rinse the mouth with bottled still water between samples.

Participants were provided with a total of 20 sorbets (4 flavours) which include five concentrations of freeze-dried Davidson plum powder (0, 5, 10, 15 and 20% *w*/*w*) over two sessions with breaks between. The sensory evaluation questionnaires were based on the sensory attributes of the Australian native ingredients [38]. The taste panel responses were obtained using Qualtrics (Qualtrics, Provo, UT, USA) platform and consisted of questions that relate to the evaluation of colour, appearance, aroma, flavour, consistency, aftertaste and characteristics of sweetness, sourness, bitterness, and astringency using a 15-point visual analogue scale [40]. The likeability, and acceptability of each of the sorbets was evaluated using a nine-point hedonic scale [41].

### 2.17. Data Analysis

All statistical analyses were conducted using IBM SPSS Statistics software (Version 28; IBM Corp., Armonk, NY, USA). For each measured parameter, descriptive statistics were calculated and reported as mean ± standard deviation (SD) to reflect central tendency and variability. To evaluate the effects of sorbet base (strawberry, raspberry, pomegranate, and Davidson plum) and the varying concentrations of freeze-dried Davidson plum powder (0%, 5%, 10%, 15%, and 20% *w*/*w*), a one-way analysis of variance (ANOVA) was performed within each flavour group. Where significant differences were observed (*p* < 0.05), post hoc comparisons were conducted using Tukey’s Honestly Significant Difference (HSD) test to determine pairwise differences among treatment means. Superscript letters in tables and figures denote statistically homogeneous subsets, with shared letters indicating no significant difference (*p* < 0.05). All experiments were conducted in triplicate, and repeatability across independent replicates was ensured.

## 3. Results

### 3.1. Macronutrient Analysis of Sorbets

The developed sorbets varied in energy (kJ) provided due to the different fruits used to produce the product (Table 1). The Davidson plum sorbet contained the highest amount of sugar and carbohydrates per 100 g.

### 3.2. Colourimetry

Fruit colour is an important aspect in the quality assessment of food and involved in consumers choice of product [42]. Colour measurement was performed on all developed sorbets (Table 2). L* represents the lightness of colour, a* indicates the amount of red (positive values) and green (negative values), and b* represents the amount of yellow (positive values) and blue (negative values). The a* and b* parameters define colour chromaticity, while the L* parameter relates to luminance, which is connected to the amount of luminous flux reflected from an object and perceived by the observer [43]. Chroma (C) is mathematically derived from the L*, a*and b* values.

The 15% and 20% sorbets appeared to be the darkest in colour when compared to the other sorbets however, there was no significant differences between them (*p* > 0.05). There was a significant difference in a* and b* values for each concentration of Davidson powder within each sorbet (*p* < 0.05), though, not significantly different between the 5, 10, 15 and 20% for the sorbet flavours. Davidson plum 0% was shown to be different in colour to all the other sorbets due to containing no fruit or Davidson plum powder, with the highest value of L and is the only sorbet that demonstrates green colour represented in the a* value.

The L*, a*and b* values for sorbets made using pomegranate as a base without Davidson plum powder was significantly different to 20% pomegranate (*p* = 0.007, *p* = 0.016, *p* < 0.001 respectively). The a* value for raspberry sorbets was significantly different for 0% and 15% (*p* = 0.004) and 5% and 15% (*p* = 0.006), but there was no significant difference between the remaining sorbets (All *p’s* > 0.05). The values for b* in raspberry sorbet were significantly different for 0% and 20% addition of Davidson plum (*p* = 0.017). The differences among the sorbet concentrations from 0% and 20% could be related to the amount of polyphenols (in particular the anthocyanins) and the composition found within each of the flavours. The Davidson plum powder containing 20% has a high amount of polyphenols resulting in a darker colour intensity than the sorbet containing no powder.

### 3.3. Melting Point

**Figure 1:**
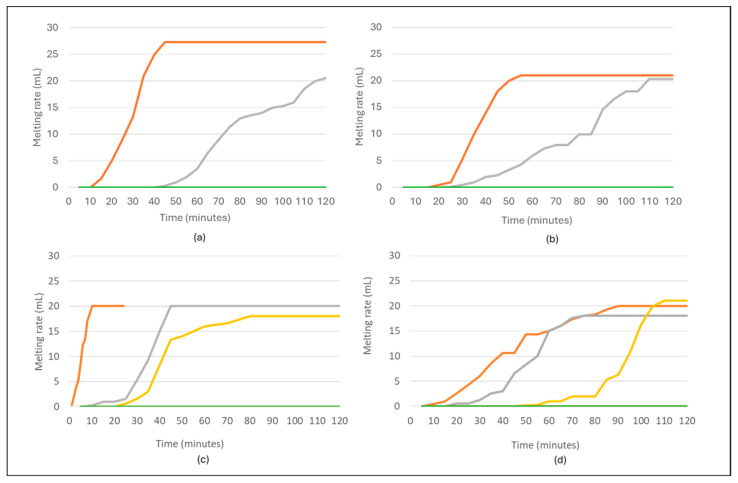
Melting rate of all sorbets at 0% (**-**), 5% (**-**), 10% (**-**), 15% (**-**) and 20% (**-**) concentrations. (**a**) Strawberry sorbet, (**b**) Raspberry sorbet, (**c**) Pomegranate sorbet, (**d**) Davidson plum sorbet. Sorbets with 10% (**-**), 15% (**-**), and 20% (**-**) Davidson plum (**a**,**b**) exhibited no melting rate and these are not individually represented in the graph.

The melting rate for each sorbet was reported in Figure 1. The sorbets containing 0% Davidson plum powder had the lowest melting rate. This may indicate the addition of Davidson plum powder at increasing ratios affects the melting kinetics of the sorbets. Pomegranate sorbet containing 5% and 10% Davidson plum were the only sorbets out of the four flavours containing Davidson plum to melt, while the sorbets containing 15% and 20% of the powder did not melt in the observed timeframe. Strawberry and raspberry sorbets containing 10%, 15%, and 20% Davidson plum powder exhibited complete resistance to melting within the timeframe of observation. This enhanced stability could be attributed to the synergistic effects between the fibre-rich Davidson plum powder and the structural properties of the fruit puree base used in both formulations. The presence of fruit puree likely contributes insoluble fibres, which in combination with the Davidson plum powder, increase the total solids content and improve water-binding capacity. This leads to the formation of a more cohesive matrix that resists melting. While pomegranate sorbet was prepared using juice rather than fruit puree, which lacks the fibre and structural components found in whole fruit. The absence of fruit puree may explain the lower structural integrity of the 10% formulation as the stabilising effect of Davidson plum powder alone at this concentration was insufficient. However, at higher concentrations (15% and 20%), the Davidson plum powder provided enough solids and fibre content to stabilise the matrix and prevent melting. Davidson plum sorbet followed a similar pattern, with only the 20% Davidson plum formulation exhibiting resistance to melting. This sorbet was made using only the powder, without any fruit puree, indicating that a high concentration of the powder is required to achieve structural stability in the absence of natural fruit fibres.

This is consistent with findings from Bilbaio et al. [44], where 3.5% of fruit powders was used in the frozen desserts. The findings indicated that the fibre content found within the fruit powders may affect the melting point. Other studies [45,46,47] have explored the utilisation of fruit and fruit powders in dairy and non-dairy frozen desserts providing insight into the potential underlying mechanisms of the melting point. Fruit and fruit powders can act as natural stabilisers due to their pectin and fibre content, helping reduce syneresis and improve the melt resistance [48]. Research into the technological formulation of frozen desserts discusses the challenges of balancing sugar, acid, and stabiliser content [49]. Fruit powder, as a functional ingredient, fits into this category by impacting both freezing and melting behaviours [50].

### 3.4. Texture Analysis

**Figure 2:**
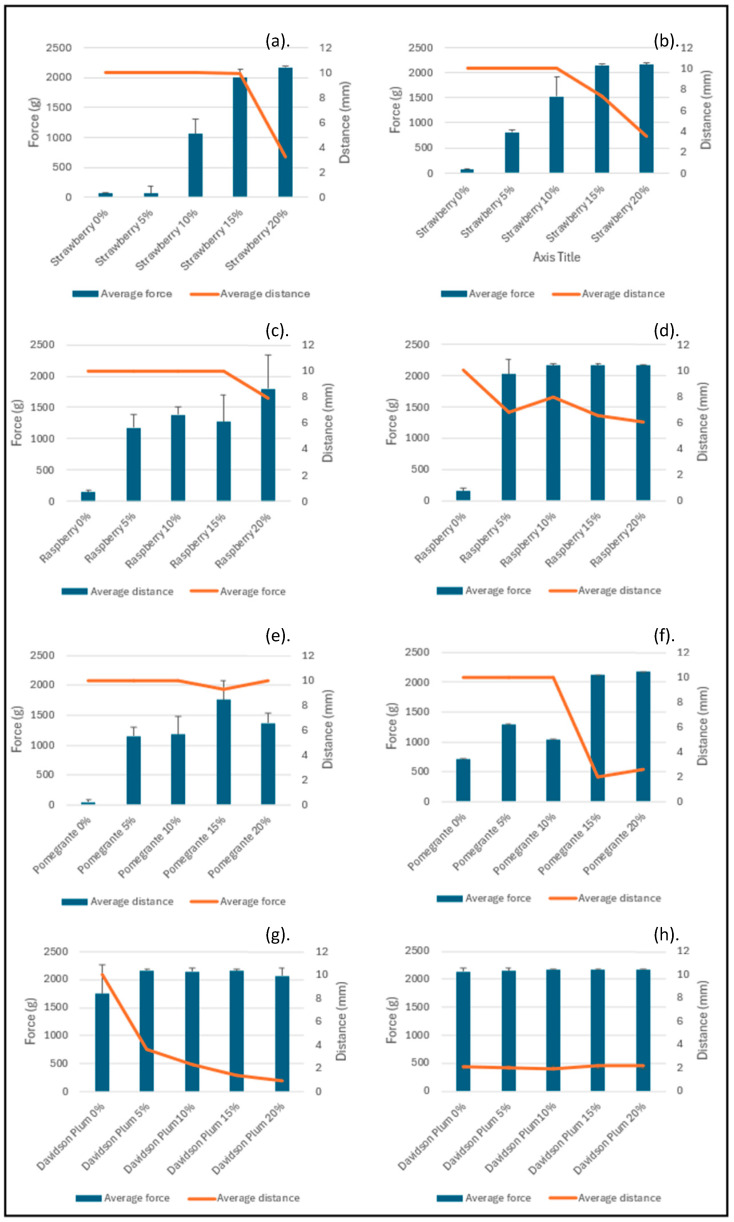
Texture properties of different sorbets during the four weeks of storage (−18 °C) (**a**) week one texture of the strawberry sorbet, (**b**) week four texture of the strawberry sorbet, (**c**) week one texture of the raspberry sorbet, (**d**) week four texture of the raspberry sorbet, (**e**) week one texture of the pomegranate sorbet, (**f**) week four texture of the pomegranate sorbet, (**g**) week one texture of the Davidson plum sorbet, (**h**) week 4 texture of the Davidson plum sorbet. Values are represented as Mean ± SD.

The texture of the sorbets was determined by measuring the force using the compression test (Figure 2). There was an increasing trend in hardness values for majority of sorbet formulations with advancing storage time and with increasing concentrations of Davidson plum powder (*p* < 0.05). The amount of Davidson plum powder incorporated within the sorbet significantly influenced the texture of the sorbet. The 20% sorbets were hardest and continued to increase in hardness over the four weeks of measurements.

### 3.5. Phytochemical Content

#### 3.5.1. Total Polyphenol and Flavonoid Analysis

The values for TPC and TFC are represented in Table 3. The TPC measured in µg gallic acid equivalents per mL (µg_GAE_/mL), increased significantly with rising fruit concentrations in all sorbet formulations (*p* < 0.05). The results demonstrated differences between and within fruit types, with Pomegranate and Davidson plum sorbets showing particularly high phenolic levels at higher concentrations. For strawberry sorbet, TPC increased from 87.19 ± 0.00 µg_GAE_/mL (0%) to 200.16 ± 0.16 (5%), 270.16 ± 0.16 (10%), 338.92 ± 0.09 (15%), and 447.29 ± 0.09 (20%), with each increase being statistically significant (*p* < 0.05). Raspberry sorbet showed a similar trend, with values increasing from 150.74 ± 0.08 (0%) to 195.10 ± 0.06 (5%), 321.73 ± 0.06 (10%), 329.87 ± 0.04 (15%), and 357.50 ± 0.04 µg_GAE_/mL at 20%. Pomegranate sorbet exhibited the highest phenolic content overall, rising from 185.88 ± 0.01 µg_GAE_/mL (0%) to 217.72 ± 0.01 (5%), 478.99 ± 0.01 (10%), 563.09 ± 0.03 (15%), and peaking at 730.74 ± 0.04 (20%). Values marked with the same superscripts indicate statistically significant differences (*p* < 0.05). Davidson plum sorbet, despite starting at a low baseline of 47.36 ± 0.02 (0%), showed a rapid increase to 108.59 ± 0.01 (5%), 326.58 ± 0.02 (10%), 442.85 ± 0.02 (15%), and 456.68 ± 0.02 µg_GAE_/mL at 20% (Table 3).

The TFC was highest in strawberry 20% (3.01 ± 0.04 µg_CE_) yet no significant difference between the sorbet flavours or concentration percentage (All p’s > 0.05). Raspberry formulations ranged from 2.16 ± 0.07 to 2.89 ± 0.03 µg_CE_/mL, with the most notable increase between 0% and 5% concentrations (2.16 ± 0.07 µg_CE_/mL (0%) to 2.73 ± 0.03 (5%)). Pomegranate sorbet had comparatively lower TFC values, starting at 0.60 ± 0.04 µg_CE_/mL (0%) and reaching 1.21 ± 0.04 µg_CE_/mL (20%). Davidson plum sorbet followed a similar trend, increasing from 0.60 ± 0.02 to 1.21 ± 0.02 µg_CE_/mL. The results indicate the flavonoid content increases with fruit concentration, although the magnitude of this increase varies by fruit type. Strawberry and Raspberry sorbets exhibited higher overall TFC values, suggesting they are richer sources of flavonoids compared to Pomegranate and Davidson plum in these formulations.

#### 3.5.2. Antioxidant Analysis

The results of FRAP analysis of different sorbets indicated that there was a significant difference between 0, 5, 10 and 15% concentration of sorbets (*p* < 0.05), however there was no significant difference (*p* = 0.116) between the 20% sorbet flavours. No significant difference between strawberry 0% (0.97 ± 0.01 µg_CE_/mL) and Davidson plum 0% (0.09 ± 0.00 µg_CE_/mL (*p* = 0.982). Strawberry sorbet showed an increase from 0.97 ± 0.01 µM_TE_ at 0% to 2.85 ± 0.05 µM_TE_ at 20%, with significant differences between each concentration (*p* < 0.05). Raspberry ranged from 1.19 ± 0.02 µM_TE_ (0%) to 2.63 ± 0.02 µM_TE_ (20%), while pomegranate increased from 0.95 ± 0.01 µM_TE_ (0%) to 2.81 ± 0.08 µM_TE_ (20%). Davidson plum exhibited the greatest increase in FRAP values (0.09 ± 0.00 µM_TE_ at 0%,2.99 ± 0.03 µM_TE_ at 20%).

The findings of CUPRAC analysis indicated a significant increase in antioxidant capacity with the increasing concentration of fruit in all sorbet formulations (*p* < 0.05). Across all four fruit types, there was a statistically significant difference in CUPRAC values between the 0%, 5%, 10%, 15%, and 20% concentrations (*p* < 0.05). Within each fruit flavour, the increase in fruit content was associated with a corresponding increase in antioxidant activity, with significant differences observed between each concentration level (*p* < 0.05). Notably, the highest CUPRAC values were recorded at 20% concentration for each flavour, with pomegranate (5.29 ± 0.05 µM_TE_) and Davidson plum (4.39 ± 0.00 µM_TE_) showing the greatest antioxidant capacities among the formulations. These results indicate that both the type and concentration of fruit incorporated into the sorbet significantly affect its antioxidant potential.

The DPPH assay results demonstrated a significant increase in radical scavenging activity with higher concentrations of fruit across all sorbet formulations. A statistically significant difference (*p* < 0.05) was observed between the 0%, 5%, 10%, 15%, and 20% fruit concentrations for each flavour. The DPPH values for Strawberry sorbet, increased from 33.79 ± 0.02 µM_TE_ at 0% to 3415.27 ± 0.04 µM_TE_ at 20%. Similarly, Raspberry exhibited a rise from 99.40 ± 0.06 µM_TE_ at 0% to 4051.41 ± 0.03 µM_TE_ at 20%, with statistically significant increases at each concentration level (*p* < 0.05). Pomegranate sorbet demonstrated the highest antioxidant activity overall, reaching 5588.08 ± 0.02 µM_TE_ at 20%. Davidson plum also showed an increase from 137.17 ± 0.03 µM_TE_ (0%) to 3292.01 ± 0.06 µM_TE_ (20%). The data shows both the fruit type and concentration significantly influence the antioxidant capacity of sorbets as measured by the DPPH assay (*p* < 0.05), with stronger activity corresponding to higher fruit incorporation.

The proanthocyanin content varied significantly across different sorbet formulations and fruit concentrations (Table 3). For the strawberry sorbets, proanthocyanin content increased from 61.68 ± 0.00 µg_CE_/mL at 0% to 99.28 ± 0.01 µg_CE_/mL at 20% and significant differences (*p* < 0.05) were observed at most concentration levels. For the raspberry sorbets, proanthocyanin values ranged from 33.74 ± 0.00 µg_CE_/mL (0%) to 91.57 ± 0.01 µg_CE_/mL (20%). The pomegranate sorbets showed the highest proanthocyanin content among all fruit types. The 0% formulation had 152.99 ± 0.00 µg_CE_/mL, increasing markedly to 196.29 ± 0.00, 233.81 ± 0.00, 288.66 ± 0.00, and peaking at 98.15 ± 0.00 µg_CE_/mL for 5%, 10%, 15%, and 20%, respectively. All increases were statistically significant, demonstrating a strong contribution of pomegranate to total proanthocyanin content. In the Davidson plum sorbets proanthocyanin content range from 115.47 ± 0.00 µg_CE_/mL (0%) to 281.44 ± 0.00 µg_CE_/mL (20%). Despite minor variation, the trend demonstrated a consistent and significant increase in proanthocyanin content with increasing fruit concentration (*p* < 0.05).

### 3.6. Sensory Evaluation

The sensory evaluation panel consisted of 12 participants (M = 5, F = 7) that were trained in aromas based on a previously developed lexicon [39]. The sorbet samples were evaluated for the sensory attributes (visual, palpatory, gustatory, acceptability and likeability). The sensory attributes of the sorbets with Davidson plum powder were performed in comparison to the control (sorbet containing no Davidson plum powder). Additional information about the sensory information is provided in the Supplementary Material. 

The strawberry 5% showed the greatest subjective score with respect to the majority of the visual parameters. The Davidson plum sorbet samples were observed to be the least visually preferred by the participants as indicative by the low subjective ratings in all visual parameters measured.

**Figure 3:**
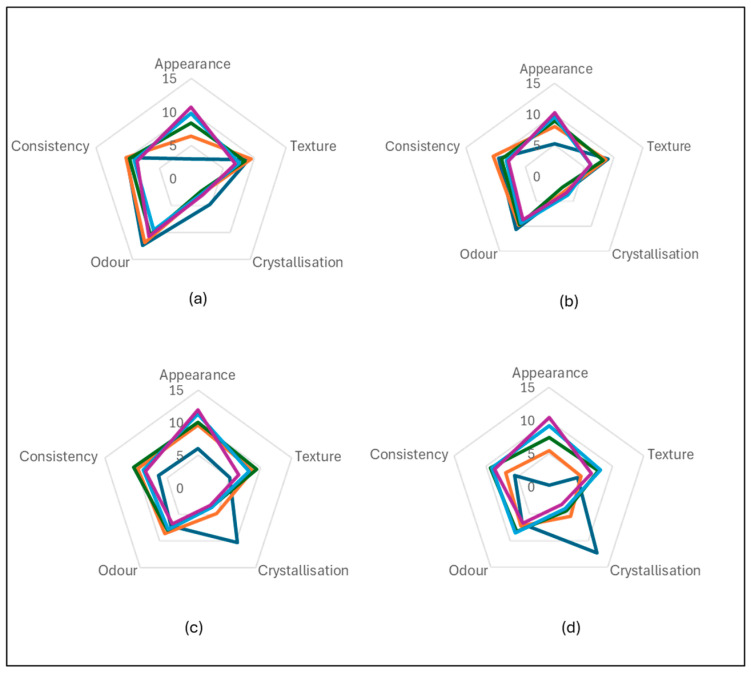
The average of the texture results for 0% (**-**), 5% (**-**), 10% (**-**), 15% (**-**), 20% (**-**) sorbet concentration flavors using the 15-point sliding scale of the 12 included semi-trained participants on the panel. (**a**) Strawberry, (**b**) Raspberry, (**c**) Pomegranate, (**d**) Davidson Plum.

Sorbets with higher fruit percentages tend to have more intense colours, stronger aromas, reduced crystallisation and thicker textures, whereas those with lower fruit concentrations are often perceived as lighter and smoother. The highest average texture score was observed for Pomegranate 20% (average = 6.75), suggesting a thicker, more cohesive texture. Lower fruit percentages (0% and 5%) consistently showed lower texture scores, with averages around 4.92 and 5.55 respectively, indicating that sorbets with less Davidson plum powder are perceived to be smoother. The presence of fruit appeared to influence texture by adding slight graininess feel to the sorbets with the addition of the Davison plum powder. Crystallization presence varied significantly across the samples, with fruit concentration influencing the degree of perceived crystallization. Davidson plum 0% and Pomegranate 0% had relatively high crystallization scores, with averages of 12.25 and 10.30, respectively. In contrast, higher fruit concentrations, such as Davidson plum 20% (average = 3.27) and Pomegranate 20% (average = 3.18), appeared to result in lower perceived crystallization (Figure 3).

**Figure 4:**
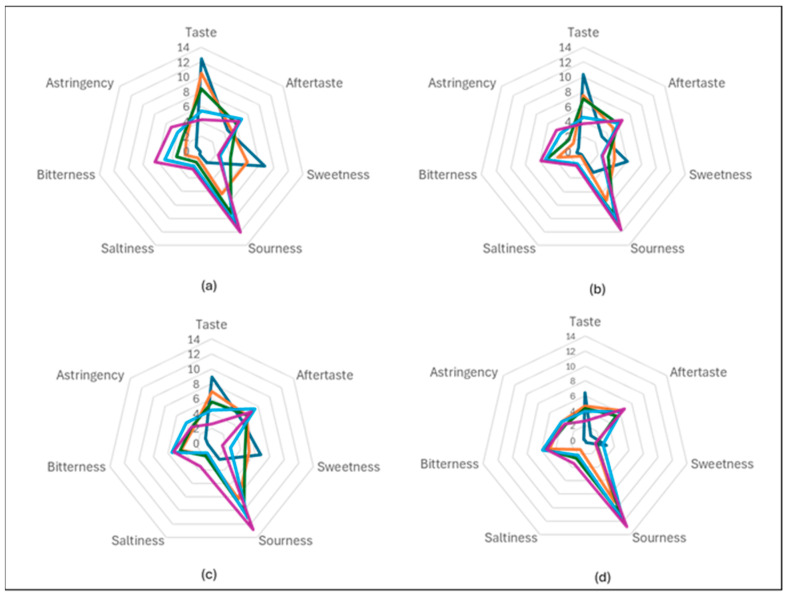
The average of the taste results for 0% (**-**), 5% (**-**), 10% (**-**), 15% (**-**), 20% (**-**) sorbet concentration flavours using the 15-point sliding scale of the 12 included semi-trained participants on the panel. (**a**) Strawberry, (**b**) Raspberry, (**c**) Pomegranate, (**d**) Davidson Plum.

All sorbets containing Davidson plum powder exhibited high levels of sourness, especially noticeable at the 20% level. Sweetness was generally low in these samples, and bitterness and saltiness were minimal across all concentrations. Strawberry and raspberry sorbet had the highest score for taste and sweetness. In contrast, the Davidson plum appeared to be the least sweet at all concentration levels (Figure 4). The inclusion of Davidson plum powder in the sorbet formulations resulted in consistently high levels of sourness across all concentrations, with the 20% concentration demonstrating particularly pronounced sour notes. This characteristic was notably more intense than the other sorbet flavours, contributing to a tart and tangy taste profile. In comparison, sweetness levels were generally low in the Davidson plum sorbet samples, as they did not reach the sweetness levels of the other fruit-based sorbets. This trend was observed at all concentration levels where Davidson plum sorbet remained tartier and sourer, lacking the natural sweetness typically associated with more conventional fruit sorbets such as strawberry and raspberry. Furthermore, the levels of bitterness and saltiness in the Davidson plum sorbet were minimal across all tested concentrations, which allowed the sourness to be the dominant sensory attribute, without significant interference from other flavours.

**Figure 5:**
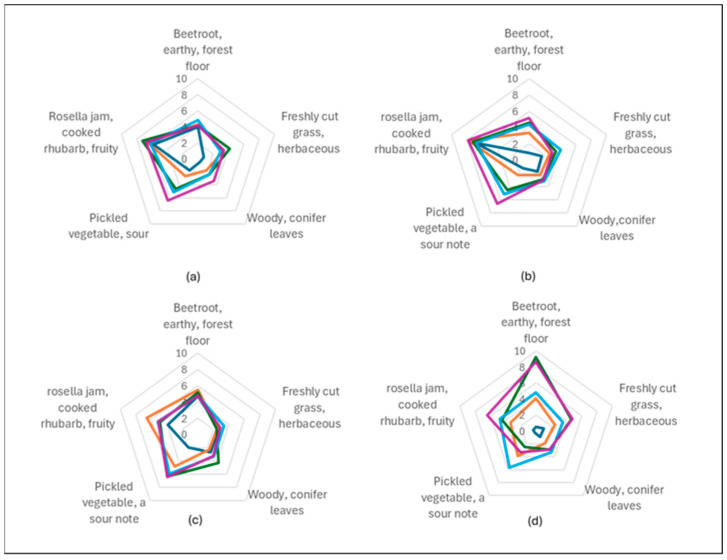
The average of the aroma results for 0% (**-**), 5% (**-**), 10% (**-**), 15% (**-**), 20% (**-**) sorbet concentration using the 15-point sliding scale of the 12 included semi-trained participants on the panel. (**a**) Strawberry, (**b**) Raspberry (**c**) Pomegranate, (**d**) Davidson plum.

Aroma assessments revealed distinct aromatic profiles across the four sorbet flavours, with notable differences in intensity and quality based on fruit type. Raspberry and strawberry sorbets received the highest overall aroma intensity ratings. Panellists most frequently associated these samples with the pleasant and recognisable aromas of rosella flowers, jam, and cooked rhubarb, indicating a strong alignment with sweet, fruity, and floral sensory notes. In contrast, Davidson plum sorbet, which received a lower overall aroma intensity rating was rated highest for fresh-cut beetroot and earthy aromas, suggesting a more root-like and grounding aromatic profile that differs from the typical fruity character of the berry sorbets. The aroma of the 20% pomegranate, strawberry and raspberry sorbet was characterised by descriptors such as pickled vegetables, a subtle sour note, and some fruity undertones. The 20% Davidson plum sorbet showed the highest aroma rating for pickled vegetable and sour notes among all samples, further reinforcing its distinctively tart and fermented aromatic qualities. These findings indicate that the berry-based sorbets (raspberry and strawberry) were perceived to have more prominent and appealing aromatic profiles (Figure 5).

**Figure 6:**
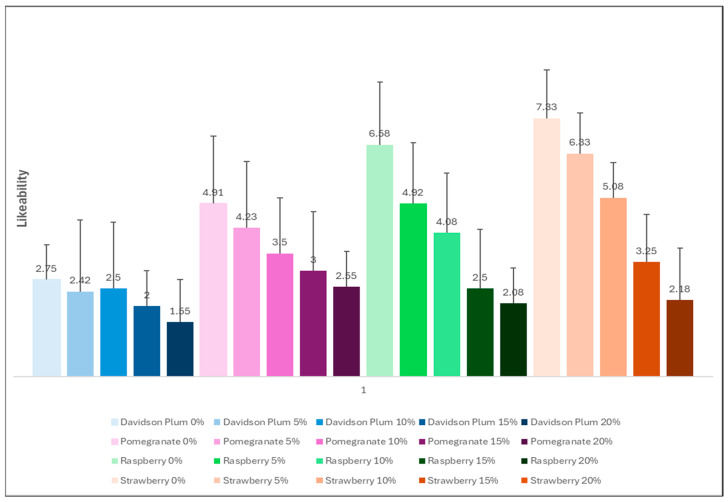
The average of the likeability results using the 9-point hedonic scale of the 12 included semi-trained participants on the panel. All values are represented as Mean ± SD.

The acceptability and likeability of the control and strawberry 5% were similar in value and above 60% (Figure 6). This was followed by strawberry 10% and thereafter raspberry with regards to acceptability and likeability values. Given this, the strawberry 5% had the greatest subjective ratings, outperforming the control in most of the sensory parameters assessed. Scores of 6 or above generally suggest that a product is liked and therefore acceptable for most purposes [51]. Although the native Australian Davidson plum is often incorporated into various food products like jams, sauces, and beverages, and is valued for its potential to improve overall health and well-being, this is one of the first studies to our knowledge that has used Australian native Davidson plum to develop and test a sorbet for sensory attributes, likeability and acceptability.

## 4. Discussion

Australian native plants offer a diverse and underexplored source of bioactive compounds with significant potential as a functional food. These endemic species have evolved in challenging environmental conditions, leading to the development of unique phytochemical profiles including high levels of antioxidants, polyphenols and anthocyanins [4,5]. Despite their nutritional and medicinal potential, the commercial utilisation of native Australian ingredients remains relatively unexplored, and their incorporation into modern food systems is still in its infancy [5,9]. The growing global demand for functional foods presents an opportunity to integrate native ingredients with high phytochemical content into innovative product development [5].

Frozen desserts such as sorbet may offer an ideal platform for delivering phytochemical-rich ingredients. Sorbet is produced and stored at low temperatures, helping to preserve bioactive compounds such as anthocyanins and flavonoids [52]. In this study, Davidson plum powder was used to develop several sorbets and examine the physicochemical, antioxidant and organoleptic properties as a functional food application.

The variation in energy content (kJ) among the developed sorbets can be attributed to the differing nutritional profiles of the fruits used (Table 1). Each fruit contributes unique levels of natural sugars, carbohydrates, and fibre, which directly impact the overall energy value of the final product. The inclusion of Davidson plum powder enhanced the dietary fibre content of the sorbet compared to the 0% fruit variations. This increase in fibre may offer additional benefits such as improving the textual properties due to their ability to increase water retention capacity [53].

The colour analysis of the sorbets indicates that the concentration of Davidson plum powder has a strong effect on the hue and chromatic intensity of the final product (Table 2). The differences in colour parameters, especially between the sorbets with and without Davidson plum powder, highlight the role of polyphenolic composition in shaping the colour profile of fruit-based sorbets. These results underscore the importance of considering both the sensory and visual appeal of sorbet products when developing formulations with Davidson plum powder. The results from the melting rate measurements show that the inclusion of Davidson plum powder impacts the melting behaviour of the sorbets (Figure 1). Sorbets without Davidson plum powder (0%) had the highest melting rate, indicating that the sorbets without the powder melted more quickly compared to those with the added fruit powder. As the concentration of Davidson plum powder increased, there was a noticeable increase in resistance to melting, with sorbets containing 15% and 20% Davidson plum powder being completely resistant to melting. The findings emphasise that the addition of Davidson plum powder influences the colour and melting behaviour of sorbets and plays a significant role in determining the textural properties (Figure 2). The results suggest that the effectiveness of Davidson plum powder in enhancing melt resistance is concentration-dependent and influenced by the type of fruit base used in the formulation. Fruit puree-based sorbets (strawberry and raspberry) required lower concentrations to achieve melting resistance, likely due to their existing fibre and pectin content, whereas juice-based or powder-only sorbets (pomegranate and Davidson plum) needed higher concentrations of Davidson plum powder to attain similar stability. This highlights the functional potential of Davidson plum powder as a technological ingredient in frozen dessert formulation.

The texture profile of the sorbets was quantitatively evaluated using a compression test, revealing significant effects of both storage duration and Davidson plum powder concentration on sorbet hardness. The freezing and thawing cycles during storage could cause ice crystal growth, making the sorbet harder and higher concentrations of the powder mean more solid particles, which can create a firmer matrix within the sorbet [52]. This could also be due to the different starting compositions of the fruits used for the preparation (pureed frozen fruit and juice.

Egg whites are often used to improve the texture of sorbet, yet this addition can also result in the interaction of egg proteins with the other phytochemicals found in fruit. The newly formed protein-phytochemical (polyphenols mainly) complex resulting in the reduced antioxidant capacity of the final product [54,55]. These complexes were also shown to alter the plasma kinetic profile of polyphenols, potentially affecting their overall absorption [56].

The different fruit bases play a role in the total amount of polyphenol and flavonoid content (Table 3). Fruits such as blueberries, strawberries, and raspberries are rich in polyphenols and flavonoids, particularly anthocyanins [57]. These differences seen in the sorbets can be attributed to the varying types and concentrations of polyphenols and flavonoids naturally present in each fruit and are influenced by factors like growing conditions, fruit maturity, and processing methods [58]. The TPC of all sorbet formulations increased with rising fruit concentrations, indicating a positive correlation between fruit inclusion and polyphenol content (Table 3). This trend was consistent across all fruit types, although the extent of increase varied, showing differences in the phenolic composition of each fruit. Notably, Pomegranate sorbets demonstrated the highest TPC values at higher concentration levels, emphasising their potential as rich sources of phenolic compounds. In contrast, the TFC showed a different pattern. While an increasing trend with higher fruit content (addition of the Davidson plum) was observed, these changes were not statistically significant across the fruit types and concentrations. Among the sorbets, strawberry at 20% Davidson plum powder recorded the highest TFC, suggesting that strawberry may be a more flavonoid-dense fruit in this formulation context. Raspberry sorbets also demonstrated relatively high TFC values, especially between the 0% and 5% levels. Pomegranate and Davidson plum sorbets showed comparatively lower TFC values across all concentrations. Despite their strong performance in TPC, these results indicate that their phenolic profile is likely composed of compounds other than flavonoids.

The FRAP values of sorbets (Table 3) increased with higher fruit concentrations, indicating enhanced antioxidant capacity which suggests the antioxidant capacity of sorbets is positively associated with fruit content [59]. This is consistent with findings that higher concentrations of fruit in food products lead to increased antioxidant activity. The variation in FRAP values among different fruit types, even at equivalent concentrations, highlights the influence of the specific phytochemical composition of each fruit on its antioxidant capacity [60]. The CUPRAC assay quantifies the reducing power of antioxidants, a higher concentration of these compounds results in greater measured antioxidant capacity. The highest CUPRAC values were recorded at the 20% fruit concentration for each sorbet flavour (Table 3), with pomegranate and Davidson plum exhibiting the greatest antioxidant capacities among the formulations. Pomegranate is rich in ellagitannins such as anthocyanins, which are highly effective reducing agents in the CUPRAC assay [60] while Davidson plum contains flavone glycosides, anthocyanins, and phenolic acids in high amounts, contributing significantly to radical scavenging and reducing activity [61]. The DPPH assay results indicate that the antioxidant capacity of sorbet formulations, as measured by radical scavenging activity, increased with rising fruit concentrations (Table 3). These findings suggest a strong positive correlation between fruit content and antioxidant potential, supporting the role of fruit-derived bioactive compounds in enhancing the functional properties of frozen desserts. The type of fruit used plays a role in determining the extent of this enhancement, with pomegranate, raspberry, and Davidson plum demonstrating the highest antioxidant activities [62]. Davidson plum sorbet at 0%, while containing its own bioactive compounds, displayed lower antioxidant capacity due to the absence of added fruit content.

Both pomegranate and berries are rich in polyphenols, but the specific types and amounts differ, with each having unique profiles that contribute to their potential health benefits [62,63]. The fruits used in this study were snap-frozen, and the juice was extracted and bottled, therefore processing variation (variation in overall food matrix composition) due to factors such as growing conditions, ripeness, and storage can potentially affect the overall antioxidant composition [64,65].

The variations of the all the sorbet flavours highlight the functional potential of different fruits when incorporated into frozen desserts. Sorbets made with puree bases offer better texture and melting stability even in the absence of stabilisers. On the contrary, juice-based or powder-only formulations benefit significantly from the inclusion of functional ingredients such as Davidson plum powder to compensate for their lack of natural stabilising agents.

The large differences in key indicators among the four 0% Davidson plum sorbets result from the intrinsic properties of each fruit, their format (juice, puree, or powder), and their individual contributions to the sorbet matrix. These findings highlight the importance of carefully selecting and balancing fruit ingredients in frozen dessert formulations to achieve desirable physicochemical and functional properties.

Based on the physicochemical, antioxidant, and sensory results obtained in this study, Davidson plum powder demonstrates promising characteristics for commercial application in frozen desserts. Sorbets with 5–10% Davidson plum powder exhibited a slower resistance to melting, a desirable trait for maintaining product integrity during storage and consumption. Additionally, the sorbets showed enhanced antioxidant capacity with increasing Davidson plum content indicating strong functional food potential. The strawberry sorbet with 5% Davidson plum was well received in sensory evaluations, highlighting both acceptability and visual appeal.

To the best of our knowledge, this is the first study to explore the sensory attributes, likeability, and acceptability of an Australian Native plant, Davidson plum in sorbet products. While Davidson plum is commonly used in a variety of food products, such as jams, sauces, and beverages, its application in frozen desserts such as sorbets is novel. The positive response to the strawberry 5% and 10% formulation demonstrates the potential for Davidson plum to be successfully integrated into frozen desserts, enhancing their sensory qualities while maintaining high consumer acceptability.

## 5. Conclusions

The strawberry and raspberry sorbet with 5% and 10% Davidson plum concentrations was well tolerated and contains moderate amount of phenolic compounds. Variations in TPC and antioxidant levels were observed between the different sorbet flavours, this could be due to the different fruit bases used for the sorbet. Pomegranate sorbet demonstrated the highest level of TPC followed by strawberry sorbet, however strawberry sorbet was the most favourable for use in a larger population group. The results demonstrated that the strawberry 10% Davidson plum functional food product is ‘liked moderately’ by consumers for taste, texture, colour and aroma. Storage time affects the products hardness and best used within the first two weeks of production. Overall, the findings suggest that Davidson plum powder, particularly at lower concentrations, can be a valuable ingredient for improving the sensory properties of sorbets and may offer health benefits. However, careful consideration of the concentration and the sensory balance of visual appeal, texture, and flavour is essential for maximising consumer satisfaction.

## Figures and Tables

**Table 1:** Nutritional profile of individual sorbets per 100 g.

Sorbet	Energy (kJ)	Protein (g)	Total Fat (g)	Carbohydrates (g)	Sugar (g)	Fibre (g)
Strawberry 0%	234.2	0.59	0.22	12.5	12.4	1.8
Strawberry 5%	301.3	0.83	0.32	14.2	12.5	5.7
Strawberry 10%	369.4	1.04	0.50	15.6	12.5	9.2
Strawberry 15%	438.25	1.28	0.65	17.4	12.6	12.8
Strawberry 20%	502.2	1.46	0.805	18.9	12.7	16.2
Raspberry 0%	291.3	0.87	0.22	14.5	9.8	3.91
Raspberry 5%	366.7	1.09	0.32	16.0	9.8	7.73
Raspberry 10%	429.2	1.30	0.48	17.4	9.9	11.2
Raspberry 15%	489.7	1.50	0.64	18.9	10.0	14.5
Raspberry 20%	548.8	1.67	0.79	20.3	10.2	17.7
Pomegranate 0%	347.9	0.30	0.00	20.2	19.4	0.36
Pomegranate 5%	408.8	0.54	0.18	21.5	19.4	4.2
Pomegranate 10%	469.0	0.77	0.52	22.7	19.1	7.78
Pomegranate 15%	534.6	1.00	0.5	24.3	19.1	11.3
Pomegranate 20%	593.3	1.19	0.67	25.6	19.1	14.6
Davidson Plum 0%	523.1	0.28	0.00	30.4	30.4	0.0
Davidson Plum 5%	696.0	1.04	0.56	29.5	29.5	12
Davidson Plum 10%	857.6	1.68	1.02	37.5	28.3	21.8
Davidson Plum 15%	986.7	2.20	1.40	39.7	27.0	30
Davidson Plum 20%	1063.0	2.50	1.67	40.4	25.3	35

**Table 2:** Colour analysis of all sorbets (L* represents the lightness of colour, a* indicates the amount of red and green, and b* represents the amount of yellow and blue). Chroma (C) is mathematically derived from the L*, a* and b* values. Means with different superscripts within each flavor are significantly different (*p* < 0.05).

Sorbet Flavor	L*	a*	b*	Chroma* (c)
Strawberry 0%	27.12 ± 5.20 ^a^	17.01 ± 3.68 ^a^	5.27 ± 1.50 ^a^	17.81 ± 3.54 ^a^
Strawberry 5%	17.86 ± 2.47 ^b^	13.86 ± 3.57 ^a^	3.45 ± 1.12 ^b^	14.24 ± 3.49 ^b^
Strawberry 10%	17.95 ± 3.32 ^b^	11.42 ± 2.67 ^b^	2.27 ± 1.09 ^b^	11.64 ± 2.63 ^c^
Strawberry 15%	17.27 ± 3.80 ^b^	9.33 ± 3.0 ^b^	1.47 ± 0.92 ^c^	9.46 ± 2.96 ^c^
Strawberry 20%	22.38 ± 5.15 ^b^	17.64 ± 5.44 ^a^	4.47 ± 1.70 ^a^	18.18 ± 5.30 ^a^
Raspberry 0%	15.90 ± 4.11 ^a^	15.00 ± 5.71 ^a^	4.13 ± 2.58 ^a^	15.55 ± 5.56 ^a^
Raspberry 5%	18.71 ± 3.11 ^b^	14.71 ± 3.45 ^a^	3.33 ± 0.92 ^a^	15.08 ± 3.37 ^a^
Raspberry 10%	14.00 ± 3.07 ^a^	10.01 ± 3.45 ^b^	1.65 ± 1.20 ^b^	10.14 ± 3.42 ^b^
Raspberry 15%	14.28 ± 3.45 ^a^	7.2 ± 3.48 ^c^	2.51 ± 1.12 ^b^	7.64 ± 3.31 ^c^
Raspberry 20%	16.54 ± 2.38 ^a^	10.30 ± 4.03 ^b^	1.89 ± 1.28 ^c^	10.47 ± 3.98 ^b^
Pomegranate 0%	21.50 ± 3.77 ^a^	10.44 ± 1.48 ^a^	3.83 ± 0.67 ^a^	11.12 ± 1.41 ^a^
Pomegranate 5%	16.48 ± 4.31 ^b^	9.80 ± 2.71 ^b^	1.73 ± 0.79 ^b^	9.95 ± 2.68 ^a^
Pomegranate 10%	17.49 ± 3.70 ^b^	10.57 ± 2.99 ^b^	1.82 ± 0.78 ^b^	10.73 ± 2.94 ^a^
Pomegranate 15%	18.53 ± 2.38 ^b^	11.74 ± 2.30 ^b^	1.42 ± 0.58 ^b^	11.83 ± 2.27 ^a^
Pomegranate 20%	15.26 ± 4.96 ^b^	6.17 ± 1.99 ^c^	−0.04 ± 1.12 ^c^	6.17 ± 1.99 ^b^
Davidson Plum 0%	44.69 ± 4.65 ^a^	−0.17 ± 0.12 ^a^	0.59 ± 0.29 ^a^	0.61 ± 0.28 ^a^
Davidson Plum 5%	25.12 ± 2.32 ^b^	16.97 ± 2.66 ^b^	2.70 ± 0.56 ^b^	17.22 ± 2.62 ^b^
Davidson Plum 10%	23.13 ± 3.84 ^b^	15.06 ± 4.25 ^b^	2.40 ± 1.01 ^b^	15.25 ± 4.20 ^b^
Davidson Plum 15%	19.77 ± 2.85 ^c^	16.68 ± 2.82 ^b^	3.14 ± 0.74 ^b^	16.97 ± 2.77 ^b^
Davidson Plum 20%	19.93 ± 4.24 ^c^	14.18 ± 2.85 ^b^	2.25 ± 0.92 ^b^	14.33 ± 2.83 ^c^

**Table 3:** Phytochemical content of all sorbet flavours. All values represented as ‘Mean ± Standard Deviation TPC—Total Phenolic Content (µg _GAE_ /mL); TFC—Total Flavonoid Content (µg/mL _CE_); DPPH—Free Radical Scavenging Activity (µM_TE_), FRAP—Antioxidant Capacity by Ferric Reducing Antioxidant Power (µM_TE_); CUPRAC—Cupric Reducing Antioxidant capacity (µM_TE_). Means with different superscripts within each flavour (^a,b,c,d,e^) are significantly different (*p* < 0.05). All values are represented as Mean ± SD.

Sorbet Flavour		Bioactive Compounds					
	TPC	TFC	FRAP	DPPH	CUPRAC	Proanthocyanins	Anthocyanins
	(μg_GAE_/mL)	(μg_CE_/mL)	(µM_TE_)	(µM_TE_)	(µM_TE_)	(µg_CE_/mL)	(µg/mL)
Strawberry 0%	87.19 ± 0.00 ^a^	2.19 ± 0.05 ^a^	0.97 ± 0.01 ^a^	33.79 ± 0.02 ^a^	1.41 ± 0.00 ^a^	61.68 ± 0.00 ^a^	5.91 ± 0.00 ^a^
Strawberry 5%	200.16 ± 0.16 ^b^	2.12 ± 0.10 ^a^	1.3 ± 0.01 ^b^	1135.11 ± 0.04 ^b^	1.97 ± 0.00 ^a^	39.51 ± 0.01 ^b^	5.77 ± 0.00 ^a^
Strawberry 10%	270.16 ± 0.16 ^c^	2.34 ± 0.07 ^a^	2.04 ± 0.04 ^c^	1894.50 ± 0.05 ^b^	3.24 ± 0.02 ^b^	45.30 ± 0.00 ^b^	6.32 ± 0.00 ^b^
Strawberry 15%	338.92 ± 0.09 ^c^	2.63 ± 0.01 ^a^	2.46 ± 0.03 ^d^	3214.49 ± 0.06 ^c^	3.46 ± 0.00 ^b^	87.91 ± 0.00 ^c^	2.37 ± 0.00 ^c^
Strawberry 20%	447.29 ± 0.09 ^d^	3.01 ± 0.04 ^b^	2.85 ± 0.05 ^e^	3415.27 ± 0.04 ^c^	4.06 ± 0.00 ^c^	99.28 ± 0.01 ^c^	4.80 ± 0.00 ^d^
Raspberry 0%	150.74 ± 0.08 ^a^	2.16 ± 0.07 ^a^	1.19 ± 0.02 ^a^	99.40 ± 0.06 ^a^	1.40 ± 0.00 ^a^	97.35 ± 0.00 ^a^	0.634 ± 0.00 ^a^
Raspberry 5%	195.10 ± 0.06 ^b^	2.73 ± 0.03 ^a^	1.80 ± 0.02 ^b^	805.11 ± 0.05 ^b^	2.29 ± 0.00 ^b^	33.74 ± 0.00 ^b^	0.067 ± 0.00 ^b^
Raspberry 10%	321.73 ± 0.06 ^c^	2.71 ± 0.06 ^a^	2.27 ± 0.03 ^c^	2769.19 ± 0.03 ^c^	3.25 ± 0.00 ^c^	59.76 ± 0.00 ^c^	0.7 ± 0.00 ^a^
Raspberry 15%	329.87 ± 0.04 ^c^	2.86 ± 0.04 ^a^	2.51 ± 0.01 ^d^	3709.48 ± 0.04 ^d^	3.61 ± 0.00 ^d^	82.89 ± 0.00 ^d^	0.133 ± 0.00 ^c^
Raspberry 20%	357.50 ± 0.04 ^c^	2.89 ± 0.03 ^a^	2.63 ± 0.02 ^d^	4051.41 ± 0.03 e	3.64 ± 0.00 ^d^	91.57 ± 0.01 ^d^	0.55 ± 0.00 ^a^
Pomegranate 0%	185.88 ± 0.01 ^a^	0.60 ± 0.04 ^a^	0.95 ± 0.01 ^a^	1119.30 ± 0.06 ^a^	1.28 ± 0.00 ^a^	86.60 ± 0.00 ^a^	0.350 ± 0.00 ^a^
Pomegranate 5%	217.72 ± 0.01 ^a^	0.83 ± 0.08 ^a^	1.27 ± 0.02 ^b^	1983.96 ± 0.05 ^a^	1.85 ± 0.00 ^a^	152.99 ± 0.00 ^b^	0.650 ± 0.00 ^b^
Pomegranate 10%	478.99 ± 0.01 ^b^	0.81 ± 0.06 ^a^	1.91 ± 0.03 ^c^	3946.05 ± 0.03 ^b^	3.25 ± 0.00 ^b^	196.29 ± 0.00 ^c^	1.15 ± 0.00 ^c^
Pomegranate 15%	563.09 ± 0.03 ^c^	0.89 ± 0.03 ^a^	2.15 ± 0.01 ^c^	4492.73 ± 0.04 ^b^	3.39 ± 0.00 ^b^	233.81 ± 0.00 ^d^	0.095 ± 0.00 ^d^
Pomegranate 20%	730.74 ± 0.04 ^d^	1.21 ± 0.04 ^b^	2.81 ± 0.08 ^d^	5588.08 ± 0.02 ^c^	5.29 ± 0.05 ^c^	288.66 ± 0.00 ^e^	1.6 ± 0.00 ^c^
Davidson Plum 0%	47.36 ± 0.02 ^a^	0.60 ± 0.02 ^a^	0.09 ± 0.00 ^a^	137.17 ± 0.03 ^a^	0.12 ± 0.00 ^a^	98.15 ± 0.00 ^a^	0.061 ± 0.00 ^a^
Davidson Plum 5%	108.59 ± 0.01 ^b^	0.75 ± 0.04 ^a^	0.70 ± 0.00 ^b^	314.10 ± 0.04 ^b^	1.06 ± 0.00 ^b^	115.47 ± 0.00 ^a^	2.116 ± 0.00 ^b^
Davidson Plum 10%	326.58 ± 0.02 ^c^	0.89 ± 0.04 ^a^	1.54 ± 0.00 ^c^	1970.04 ± 0.04 ^c^	2.12 ± 0.00 ^c^	229.49 ± 0.00 ^b^	4.115 ± 0.00 ^c^
Davidson Plum 15%	442.85 ± 0.02 ^d^	1.08 ± 0.03 ^b^	2.44 ± 0.02 ^d^	2135.04 ± 0.07 ^c^	3.63 ± 0.01 ^d^	268.46 ± 0.00 ^c^	4.97 ± 0.00 ^d^
Davidson Plum 20%	456.68 ± 0.02 ^d^	1.21 ± 0.02 ^b^	2.99 ± 0.03 ^e^	3292.01 ± 0.06 ^d^	4.39 ± 0.00 ^e^	281.44 ± 0.00 ^c^	2.87 ± 0.00 ^e^

## Data Availability

Data will be made available upon a reasonable request.

## Supplementary Materials

The following supporting information can be downloaded at: https://www.mdpi.com/article/10.3390/foods14162902/s1, Table S1: The average and SD of the aroma results for strawberry sorbet concentration using the 15-point scale of the 12 included semi-trained participants on the panel; Table S2: The average and SD of the aroma results for raspberry sorbet concentration using the 15-point sliding scale of the 12 included semi-trained participants on the panel; Table S3: The average and SD of the aroma results for pomegranate sorbet concentration using the 15-point sliding scale of the 12 included semi-trained participants on the panel; Table S4: The average and SD of the aroma results for Davidson plum sorbet concentration using the 15-point sliding scale of the 12 included semi-trained participants on the panel.

## Abbreviations

The following abbreviations are used in this manuscript:

DPPH	2,2-diphenyl-1-picrylhydrazyl
TPTZ	2,4,6-Tri(2-pyridyl)-s-triazine
FRAP	Ferric Reducing Antioxidant Power
CUPRAC	Cupric Reducing Antioxidant Capacity
TPC	Total polyphenol content
TFC	Total Flavonoid Content
