# Supplementary material for: Physicochemical and Sensory Properties of Davidson Plum (Davidsonia jerseyana) Sorbet, a Potential for New Functional Food Product
Source: Foods. 2025 Aug 21;14(16):2902. doi: 10.3390/foods14162902 (PMC12385496; doi:10.3390/foods14162902)
Supplement: Supplementary file 1 [file foods-14-02902-s001.zip › foods-3791692-supplementary.pdf]

Table S1: The average and SD of the aroma results for strawberry sorbet concentration using the 15-point sliding scale of the 12 included semi-trained participants on the panel

	Appearance	Texture	Crystallisation	Odour	Consistency	Fresh cut beetroot	Freshly cut grass	Conifer leaves	Pickled vegetable	Rosella taste	Taste	Aftertaste	Sweetness	Sourness	Saltiness	Bitterness	Astringency
Strawberry 0%	2.91±1.16	9.08±4.12	4.81±5.02	12.41±1.62	10.16±2.82	0.91±1.81	1.63±2.92	1.71±2.98	1.22±2.99	6.92±5.47	12.42±1.93	4.55±3.22	8.75±2.26	1.67±2.60	0.50±1.07	0.13±0.35	0.89±1.17
Strawberry 5%	6.33±1.50	9.41±2.64	2.72±±3.2	11.90±1.85	10.25±1.42	3.36±3.26	2.62±3.29	2.30±3.02	2.30±3.62	7.83±3.83	10.50±1.51	5.00±3.38	6.33±2.67	6.33±3.77	1.00±1.12	2.20±2.97	2.55±2.46
Strawberry 10%	8.33±1.97	8.50±3.48	2.36±2.58	10.50±2.78	9.75±2.53	4.54±3.72	3.44±4.56	2.88±2.42	4.54±5.34	7.25±3.77	8.42±1.88	6.00±3.07	4.00±1.76	9.25±3.14	1.56±2.55	3.45±3.14	3.11±3.02
Strawberry 15%	9.75±2.22	7.58±4.52	2.72±3.26	9.63±3.01	8.91±3.70	4.36±3.72	4.11±4.23	3.20±3.22	5.20±4.66	6.83±3.19	5.42±2.07	6.91±3.18	2.60±1.71	10.75±3.19	2.22±3.23	5.00±4.02	4.10±3.87
Strawberry 20%	10.75±2.67	7.00±4.51	3.00±3.29	10.75±2.67	8.50±3.48	5.18±3.63	3.00±3.64	3.00±4.14	6.63±5.64	7.83±3.13	4.25±2.26	6.50±2.50	2.33±2.29	12.08±2.91	2.62±3.02	6.40±5.04	5.20±4.21

Table S2: The average and SD of the aroma results for raspberry sorbet concentration using the 15-point sliding scale of the 12 included semi-trained participants on the panel

	Appearance	Texture	Crystallisation	Odour	Consistency	Fresh cut beetroot	Freshly cut grass	Conifer leaves	Pickled vegetable	Rosella taste	Taste	Aftertaste	Sweetness	Sourness	Saltiness	Bitterness	Astringency
Raspberry 0%	5.25±1.14	9.00±2.53	3.09±1.45	10.60±2.50	9.42±2.39	4.00±4.67	0.77±0.97	0.44±1.01	1.80±3.16	5.83±3.59	10.33±3.03	3.20±2.39	6.09±2.21	3.10±2.02	0.50±0.76	0.89±1.27	0.77±1.09
Raspberry 5%	8.00±1.30	8.75±2.47	2.90±1.58	9.75±2.95	10.41±2.14	4.27±3.14	3.30±3.76	1.77±2.91	2.66±3.6	6.50±3.83	7.50±3.32	5.09±1.90	4.30±2.87	7.20±3.79	0.75±1.39	3.54±3.56	1.70±1.58
Raspberry 10%	8.92±2.06	8.16±3.59	2.20±1.81	9.58±2.60	9.00±3.09	4.16±4.05	4.18±3.65	2.40±2.46	4.63±3.96	7.25±3.20	7.09±2.14	6.00±3.01	3.40±1.71	9.41±3.77	2.00±2.20	5.10±4.61	2.50±2.84
Raspberry 15%	9.58±1.98	6.17±3.83	3.72±3.58	9.33±2.39	8.16±3.04	4.83±2.98	3.00±2.45	2.40±2.27	5.10±5.13	6.25±3.72	4.58±2.59	6.09±1.51	2.56±3.80	10.33±2.30	1.88±4.35	5.18±3.75	3.90±1.68
Raspberry 20%	10.25±2.01	6.08±4.44	3.33±4.27	8.66±2.71	7.83±3.88	4.16±3.49	3.40±2.59	3.40±3.53	6.36±5.57	6.75±4.11	3.75±1.54	6.66±3.06	2.66±2.45	11.66±3.37	2.11±2.67	5.81±4.53	4.63±4.8

Table S3: The average and SD of the aroma results for pomegranate sorbet concentration using the 15-point sliding scale of the 12 included semi-trained participants on the panel

	Appearance	Texture	Crystallisation	Odour	Consistency	Fresh cut beetroot	Freshly cut grass	Conifer	Pickled vegetable	Rosella taste	Taste	Aftertaste	Sweetness	Sourness	Saltiness	Bitterness	Astringency
Pomegranate 0%	6.09±2.47	5.09±3.51	10.30±3.27	7.10±2.85	6.36±3.64	4.73±5.18	2.70±2.21	2.67±3.04	2.00±3.46	3.91±3.39	8.91±3.39	5.10±3.78	6.73±2.94	2.36±2.73	0.56±1.01	0.50±0.85	1.10±1.6
Pomegranate 5%	9.62±2.02	8.77±2.95	4.83±2.48	8.58±2.35	9.69±2.25	5.46±3.95	2.55±2.42	2.30±2.31	4.83±4.57	6.62±3.45	7.00±3.46	6.00±2.35	5.18±2.40	8.31±4.07	1.78±1.56	4.18±4.21	3.18±2.96
Pomegranate 10%	10.00±1.35	9.42±3.15	3.55±3.21	8.00±1.79	10.33±2.39	5.17±3.04	2.40±2.27	4.27±3.38	6.36±4.76	4.92±3.09	5.55±2.58	6.00±2.09	4.60±2.80	10.00±3.16	1.89±1.96	4.36±3.88	3.45±2.11
Pomegranate 15%	11.33±1.68	8.25±2.98	3.64±3.04	7.83±2.35	8.83±2.84	4.75±3.17	3.36±3.80	3.36±2.26	6.00±5.11	5.16±2.50	4.50±2.54	7.41±2.63	2.60±2.15	11.16±2.98	1.44±2.11	5.45±5.49	4.36±3.93
Pomegranate 20%	11.92±2.02	6.58±4.29	3.18±3.34	6.75±2.80	8.42±4.06	5.25±3.44	3.80±2.90	3.27±2.84	6.42±5.32	5.08±2.93	2.58±2.53	6.82±2.97	1.44±1.58	12.83±2.25	3.44±3.24	5.27±5.91	3.5±4.84

Table S4: The average and SD of the aroma results for Davidson plum sorbet concentration using the 15-point sliding scale of the 12 included semi-trained participants on the panel

	Appearance	Texture	Crystallisation	Odour	consistency	Fresh cut beetroot	Freshly cut grass	Conifer	Pickled vegetable	Rosella taste	Taste	Aftertaste	Sweetness	Sourness	Saltiness	Bitterness	Astringency
Davidson Plum 0%	0.30±0.00	4.50±2.81	12.25±2.60	6.92±2.39	5.42±3.65	0.50±1.07	1.00±1.12	0.89±1.83	0.25±0.71	0.38±1.06	6.42±3.48	1.00±1.84	2.91±2.07	0.30±0.67	0.00±0.00	0.00±0.00	0.20±0.44
Davidson Plum 5%	5.5±2.07	5.08±3.15	5.54±2.98	7.25±2.63	6.91±2.91	4.08±3.37	2.50±2.72	1.91±2.02	3.83±4.24	3.36±3.50	4.66±2.84	6.41±3.34	1.44±1.24	9.16±4.63	1.37±2.39	4.90±5.11	4.00±3.79
Davidson Plum 10%	7.42±1.68	7.80±2.96	4.41±3.20	8.33±1.83	9.25±2.42	4.67±3.47	4.67±3.47	2.90±1.91	2.45±2.11	4.50±4.36	4.36±3.56	5.33±3.03	1.63±1.19	11.00±3.74	2.67±4.03	5.45±5.11	3.36±4.18
Davidson Plum 15%	9.25±1.79	8.08±3.52	4.09±3.90	8.58±2.90	9.08±2.15	4.83±3.48	3.50±2.88	3.27±3.17	5.66±4.90	4.75±3.09	3.91±3.43	6.16±3.26	2.44±2.31	11.91±2.98	2.20±3.21	5.81±4.84	4.00±4.18
Davidson Plum 20%	10.41±2.15	6.66±3.85	3.27±3.47	6.73±2.76	8.66±3.75	4.75±3.25	2.91±2.81	3.27±2.90	6.42±5.65	5.08±3.40	2.58±1.93	6.82±3.89	1.44±1.24	12.83±2.52	3.44±3.64	5.28±5.18	3.50±3.03
